# Integrated peripheral immune profiling reveals B-cell dysregulation and CD8 effector signatures in chronic inflammatory demyelinating polyneuropathy

**DOI:** 10.3389/fimmu.2026.1888882

**Published:** 2026-07-24

**Authors:** Hyunjin Kim, Jinhui Chun, Do Hyeon Cha, Jeong Seok Lee, Dayoung Seo, Wangyong Shin, Inhye Jang, Jihong Ryu, Lynkyung Choi, Jinhee Kim, Young-Min Lim, Changuk Chung, Sang-Hyun Hwang, Eun-Jae Lee

**Affiliations:** 1 Department of Neurology, Asan Medical Center, University of Ulsan College of Medicine, Seoul, Republic of Korea; 2 Graduate School of Medical Science and Engineering, Korea Advanced Institute of Science and Technology, Daejeon, Republic of Korea; 3 Biomedical Research Center, Asan Institute for Life Sciences, Asan Medical Center, Seoul, Republic of Korea; 4 Department of Laboratory Medicine, Asan Medical Center, University of Ulsan College of Medicine, Seoul, Republic of Korea

**Keywords:** B cells, CD8 T cells, chronic inflammatory demyelinating polyneuropathy, inflammasome, multiple sclerosis, peripheral immune profiling, single-cell RNA sequencing

## Abstract

**Background:**

Chronic inflammatory demyelinating polyneuropathy (CIDP) is an immune-mediated peripheral neuropathy with heterogeneous and often incomplete responses to current immunotherapies, but the underlying immune basis remains poorly defined. Although CIDP shares features of immune-mediated demyelination with multiple sclerosis (MS), the two diseases affect distinct anatomical compartments and exhibit divergent therapeutic responses, suggesting fundamentally different underlying immune programs. Here, we address this gap by defining the peripheral immune architecture of CIDP using an integrated, multi-modal approach.

**Methods:**

Peripheral blood was obtained from 20 patients with CIDP and 20 age- and sex-matched healthy controls. Single-cell RNA sequencing was performed in a discovery subset and integrated with publicly available MS peripheral blood datasets to provide a cross-disease reference framework. The single-cell analysis was designed as an exploratory discovery step to identify candidate immune signatures. Transcriptomic, pathway, and ligand–receptor analyses were complemented by cytokine profiling and flow-cytometric validation in the full cohort.

**Results:**

CIDP exhibited broad inflammatory activation with preferential enrichment of type I interferon and inflammasome-related programs compared with MS. Despite reduced B-cell frequencies, CIDP showed transcriptional enrichment of germinal center–associated programs, indicating a dissociation between cell number and activation state. In parallel, CD8 effector T cells demonstrated enhanced cytotoxicity and cytoskeletal remodeling programs, supported by increased expression of actin-regulatory genes and strengthened intercellular signaling interactions. In contrast, MS showed greater enrichment of integrin–talin–vinculin signaling pathways in B cells and CD4 T-cell subsets, consistent with trafficking-related immune mechanisms. Together, these findings indicate a coordinated immune axis linking B-cell dysregulation and cytotoxic CD8 T-cell activation in CIDP.

**Conclusions:**

Integrated peripheral immune profiling identified candidate CIDP-associated immune signatures including dysregulated B-cell activation despite numerical reduction and a prominent cytotoxic CD8 T-cell program within a type I interferon– and inflammasome-skewed inflammatory milieu. These findings provide an exploratory framework for understanding peripheral immune dysregulation in CIDP and warrant further translational studies in larger, treatment-stratified cohorts.

## Introduction

Chronic inflammatory demyelinating polyneuropathy (CIDP) is an immune-mediated disorder of the peripheral nervous system characterized by progressive motor and sensory impairment driven by peripheral nerve demyelination (1). Although treatments such as intravenous immunoglobulin, corticosteroids, and plasmapheresis are often effective, a substantial proportion of patients remain only partially responsive or experience relapses, indicating persistent immune activity and underscoring heterogeneity in pathogenesis (2). This highlights a critical unmet need to define the underlying immune mechanisms in CIDP. Multiple immune mechanisms involving T cells, B cells, autoantibodies, and complement have been proposed (2), yet the specific immune cell subsets and molecular pathways driving CIDP remain poorly defined.

Multiple sclerosis (MS) is a related autoimmune demyelinating disease affecting the central nervous system. In MS, B cells display increased activation and clonal expansion, often accompanied by CD4 T-cell activation through dysregulated interferon-γ signaling (3, 4). The clinical success of B-cell depletion with ocrelizumab and type I/II interferon modulation with interferon-β highlights the relevance of these pathways (5, 6). Histopathological studies describing blood-brain barrier disruption and perivascular immune infiltration (7, 8), along with the efficacy of natalizumab in reducing leukocyte trafficking (9), further underscore the central role of immune cell migration and activation. Together, these findings define a well-characterized immune framework in MS that can serve as a reference for understanding immune dysregulation in other demyelinating diseases.

Despite their shared hallmark of immune-mediated demyelination, MS disease-modifying therapies have not shown consistent benefit in CIDP (10, 11), suggesting that peripheral and central myelin injury may be driven by distinct immune programs. This clinical divergence underscores a critical gap in our understanding of CIDP-specific immunopathogenesis. Comparative single-cell approaches integrating CIDP with MS as a reference framework provide an opportunity to delineate disease-specific immune states and to identify mechanisms underlying heterogeneous therapeutic responses.

In this study, we integrated single-cell transcriptomics with pathway analysis, ligand–receptor mapping, cytokine profiling, and flow-cytometric validation to define the peripheral immune architecture of CIDP and to contextualize it against MS as a cross-disease reference. Our goal was to identify mechanisms underlying heterogeneous therapeutic responses, with potential implications for biomarker development and targeted immunotherapy in CIDP.

## Methods

### Participants

We enrolled consecutive patients with CIDP who visited Asan Medical Center between March 2019 and July 2020 (**Supplementary Table 1**). The study was approved by the Institutional Review Board of Asan Medical Center (No. 2018-0653) and conducted in accordance with the Declaration of Helsinki. Written informed consent was obtained from all participants. Inclusion criteria were adults with a definite diagnosis of CIDP according to the 2010 EFNS/PNS criteria (12).

Age- and sex-matched healthy controls (HCs) were recruited during the same period as a hospital-based control cohort. These individuals presented with non-specific headache or dizziness, had no neurological deficits, and showed no abnormalities on brain MRI or CT. Participants with conditions affecting peripheral nerve function or systemic immune status, including central nervous system disorders, diabetes mellitus, chronic alcohol use, or systemic inflammatory diseases, were excluded.

### Flow cytometry analyses

Cryopreserved peripheral blood mononuclear cells (PBMCs) were thawed and subjected to flow cytometric immunophenotyping using commercially available DuraClone reagent panels according to the manufacturer’s instructions (Beckman Coulter, Brea, CA, USA). Regulatory B-cell analysis was performed using the DuraClone IM B Cells Tube (Cat. No. B53318), and regulatory T-cell analysis was performed using the DuraClone IM Treg Tube (Cat. No. B53346). No additional antibodies were added to the commercial reagent panels. Regulatory B cells were defined as CD19^+^CD24^hi^CD38^hi^ cells. Regulatory T cells were identified as CD3^+^CD4^+^CD25^+^FoxP3^+^ cells. Representative gating strategies are shown in **Supplementary Figure 4**. Detailed information on the antibody panels, including target markers, fluorochromes, clones, and manufacturers, is provided in **Supplementary Table 4**. Data were acquired using a 10-color Navios™ flow cytometer (Beckman Coulter, Miami, FL, USA) and analyzed using Kaluza^®^ Flow Analysis Software (Beckman Coulter).

### Cytokine measurements

Serum concentrations of GM-CSF, Granzyme B, IFN-γ, IL-1β, IL-2, IL-4, IL-5, IL-6, IL-10, IL-12p70, IL-13, IL-15, IL-17A, TNF-α, and TGF-β1 were quantified at an external laboratory (Woongbee Meditech Inc., Korea) using the Human XL Cytokine Luminex^®^ Performance Base Kit (LUXLM000) and the Magnetic Luminex^®^ Performance Assay for TGF-β (LTGM00).

### Single-cell RNA sequencing

For single-cell RNA sequencing (scRNA-seq), we performed a focused analysis in a subset of samples as a hypothesis-generating approach to identify candidate disease-associated immune signatures. Cryopreserved PBMCs were rapidly thawed and washed in 10 ml X-Vivo15 media. A 5μL aliquot of the single cell suspension was counted manually in a Fuchs-Rosenthal chamber. Up to 10,000 cells were used as input. Single-cell suspensions were loaded onto the Chromium Single Cell Controller using the Chromium Single Cell 3′ Library & Gel Bead Kit v2 (both from 10X Genomics). Sample processing and library preparation was performed according to manufacturer instructions using AMPure beads (Beckman Coulter) and sequenced locally on an Illumina Nextseq 500 using the High-Out 75 cycle kit with a 26-8-0–57 read configuration.

### Single-cell data processing and integration

Raw single-cell RNA sequencing data were processed using CellRanger (v4.0) for demultiplexing, alignment to the GRCh38 reference genome, and UMI quantification, generating gene-cell count matrices. All downstream analyses were performed in R (Seurat v5.3.0). In-house PBMC scRNA-seq data from two patients with CIDP and two age- and sex-matched healthy controls were integrated with publicly available datasets consisting of three relapsing–remitting Multiple sclerosis (MS) samples (GSE133028) and three additional HC samples (GSE149689) (13, 14), yielding a total of 10 datasets. To ensure comparability across batches and platforms, only genes detected across all datasets were retained.

Quality control was applied uniformly across datasets. Cells were removed if they had <500 or >2,500 detected genes, <1,000 or >15,000 total Unique Molecular Identifiers (UMIs), or >15% mitochondrial transcripts. Ambient RNA contamination was removed using decontX (v1.6.0), excluding cells with contamination ≥0.1, and doublets were predicted with scDblFinder (v1.22.0) and discarded (15, 16). After filtering, 23,717 high-quality cells and 11,455 genes were retained for analysis. Gene counts were log-normalized, and the top 2,000 variable genes were selected. Technical covariates including mitochondrial percentage, ribosomal percentage, decontX contamination score, and doublet score were regressed out during data scaling. We then performed PCA and used 30-dimensional FastMNN of batchelor (v1.24.0) to integrate datasets and correct for donor-level batch effects (17). The resulting batch-corrected embeddings were used to generate a Uniform Manifold Approximation and Projection (UMAP) representation.

To obtain robust and reproducible cell-type annotations, we first used the Azimuth Human PBMC Cellular Indexing of Transcriptomes and Epitopes sequencing (CITE-seq) reference and performed anchor-based label transfer and query mapping to assign initial cell identities (18). We then performed fine clustering using the FastMNN-corrected dimensions, applying Seurat’s FindNeighbors and FindClusters with a high resolution (10.0) to increase granularity. Each cluster was subsequently re-annotated using a majority-voting strategy, assigning the most frequently transferred Azimuth label within each cluster as its final identity.

To validate annotation accuracy, we examined canonical marker gene expression of each annotated cell type. Additionally, we generated pseudobulk expression profiles for the annotated cell types and performed pairwise Pearson correlation and hierarchical clustering with average linkage method to assess the biological relatedness of immune cell lineages.

### Immune signature and pathway analysis

To characterize immune activation states across disease conditions, module scores were calculated using Seurat’s AddModuleScore function with curated immune-related gene signatures (**Supplementary Table 2**) (19), including type I/II interferon responses, pro-/anti-inflammatory signaling, inflammasome activation, and cytotoxicity (20, 21). To assess differences in immune activation between CIDP, MS, and HC, we performed statistical comparisons within each cell type for each immune signature. For each module score, we first applied a Kruskal–Wallis test across the three groups. If the global test was significant (*P* < 0.05), we conducted *post-hoc* Dunn’s tests with Bonferroni adjustment to identify pairwise differences. We defined a pairwise comparison as “significantly different” if it met both of the following criteria: (i) either the Kruskal–Wallis test or the Dunn *post-hoc* test was statistically significant (adjusted *P* < 0.05), and (ii) the absolute difference in median module score exceeded 0.02, ensuring that statistically significant differences also reflected meaningful biological effect sizes.

### Cell cycle module scoring

To evaluate proliferative activity across disease conditions, we calculated S phase module scores using the Seurat-curated cell cycle gene set (cc.genes.updated.2019) with the AddModuleScore function. Statistical comparisons were performed within each cell type, following the same framework as the immune signature analysis. Specifically, Kruskal–Wallis tests were applied to compare S phase scores across CIDP, MS, and HC, and *post-hoc* Dunn’s tests with Bonferroni correction were conducted when the global test was significant (*P* < 0.05) to identify pairwise differences.

### Pathway activity enrichment analysis using gene set variation analysis

We performed GSVA framework (v2.2.0) to quantify pathway activity at the single-cell level across different conditions (22). KEGG MEDICUS gene sets from the MSigDB C2 collection were used to compute GSVA enrichment scores for each pathway in every cell. To compare pathway activity across disease groups, we analyzed GSVA scores within each cell type and performed pairwise Wilcoxon rank-sum tests for the following contrasts: CIDP vs HC, MS vs HC, and CIDP vs MS. For each pathway × cell type combination, we calculated the median difference in GSVA scores as an effect size and applied Benjamini–Hochberg FDR correction across all tests.

To identify pathways that exhibited the largest overall differences between CIDP and HC, we aggregated effect sizes across all cell types. For each pathway, we calculated the sum of absolute median differences to quantify the overall magnitude of change. Pathways with positive signed sums were considered higher in CIDP, and those with negative signed sums were considered lower. We then selected the top 30 pathways with the highest activity in CIDP and the top 15 pathways with the lowest activity based on the aggregated effect size. These were visualized using bubble plots along with MS vs HC and CIDP vs MS results on the same pathways, where bubble color represented the direction and magnitude of the median difference, and bubble size encoded FDR-adjusted *P*-values.

To further explore graph network of top differential pathways in CIDP-HC comparison, we constructed and visualized pathway–pathway overlap network based on shared genes among the 30 CIDP-upregulated and 15 CIDP-downregulated. Clustered pathways of CD8 effector T cells revealed core genes (e.g., *ACTR2, ACTR3, ARPC1B*) related to actin-remodeling functionality, and the expression of these genes were compared across CIDP, MS, and HC using Kruskal–Wallis tests followed by *post-hoc* Dunn tests.

### Cell–cell interaction analysis

Cell–cell communication was inferred on a per-donor basis using 1,000 permutations using CellPhoneDB (v5.0.1) (23). and significant ligand–receptor interactions were defined as *P* < 0.05. For each donor, we counted the number of significant (n_sig) and tested (n_total) interactions for every sender–receiver cell-type pair. To compare how frequently cell types engaged in significant interactions across disease groups (CIDP vs HC, MS vs HC, CIDP vs MS), donor-level counts were aggregated within each group and two-sided Fisher’s exact tests were applied to 2×2 tables of n_sig vs n_nonsig. The difference in the proportion of significant interactions (n_sig/n_total) was used as the effect size, and Benjamini–Hochberg FDR correction was applied. Results were visualized as heatmaps.

To examine condition-specific differences in ligand–receptor signaling, we compared CellPhoneDB aggregate mean interaction scores between groups for each sender–receiver pair. Interactions significant in only one group were considered present exclusively in that condition and were assigned the corresponding mean score, whereas interactions significant in both groups were directly compared using the difference in mean scores (e.g., CIDP − HC) to assess directionality and magnitude of change. These results were visualized using bubble plots, where red and blue bubbles denoted interactions observed only in CIDP or HC, respectively, and outlined bubbles indicated interactions significant in both groups with color reflecting the direction and magnitude of the difference. Circle size represents the statistical significance of each ligand–receptor interaction, quantified as the −log_10_ of the corresponding P-value. For interactions significant in both conditions, the smaller of the two −log_10_ P-values was used to determine bubble size. Ligand–receptor pairs were then ranked by the absolute magnitude of their differences across sender–receiver contexts to highlight the most strongly dysregulated intercellular communication events across disease conditions.

### B cell subclustering and differential testing

To investigate disease-associated heterogeneity within the B cell subpopulation, we isolated all B cells from the integrated dataset and performed subset-specific reclustering. For each B cell subset (B Naïve and B Memory), we recomputed the nearest-neighbor graph using the FastMNN embeddings and applied Seurat’s FindClusters with a resolution of 0.5, which yielded discrete subclusters within both populations.

In the B Naïve subset, several subclusters displayed disease-biased distributions, including clusters enriched in CIDP, MS, or HC. To characterize the transcriptional programs underlying these clusters, we applied Seurat’s FindAllMarkers (Wilcoxon rank-sum test, log2FC > 0.25, positive markers only) to identify differentially expressed genes (DEGs).

We then focused on the CIDP-enriched B Naïve subcluster and performed over-representation analysis (ORA) to interpret its molecular signature. gprofiler2 (v0.2.3) was used to examine enrichment of Gene Ontology Biological Process (GO: BP) terms (24), and enrichR (v3.4) was applied with curated B Naïve transcriptional programs to perform a custom ORA (e.g., early germinal center programs) (25).

To further interpret the biological diversity within the Naïve B cell population, we next sought to define its potential subtypes. We observed that the B cell population within PBMCs from patients with the disease of interest exhibited a distinct clustering pattern compared to healthy controls. Based on established literature, we identified markers characterizing B-1, B-2, and regulatory B (Breg) cells (26), as well as activation stage–specific markers of Germinal Center B cells (27). Additionally, we incorporated findings from single-cell resolution marker studies of B cells (28) to comprehensively define a total of 19 subtypes. Each subtype was then ranked according to its degree of overlap with positive log-fold change differentially expressed genes (DEGs) identified in our dataset. These analyses collectively revealed functional pathways and regulatory programs associated with CIDP-specific Naïve B cell states. These analyses revealed functional pathways and regulatory programs associated with CIDP-specific B Naïve states.

## Results

### Cohort characteristics and single-cell profiling

**Figure 1:**
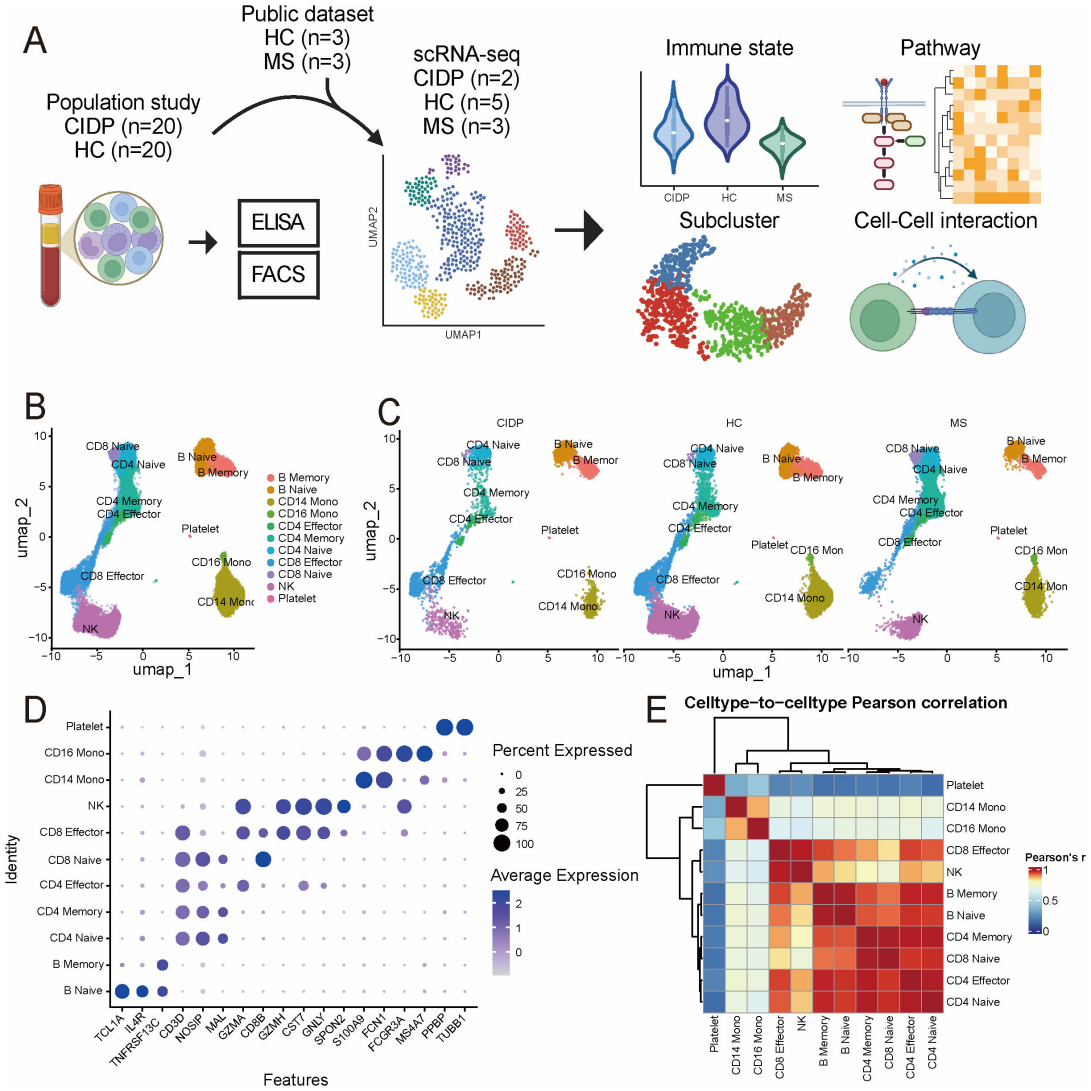
Overview of scRNA-seq profiles from CIDP, HC, and MS PBMCs. **(A)** Cohort design and data analysis workflow. Peripheral blood was collected from CIDP and matched HC participants for cytokine profiling and flow cytometry. A subset (CIDP *n* = 2, HC *n* = 2) underwent scRNA sequencing, and the data were integrated with publicly available MS (*n* = 3) and HC (*n* = 3) datasets to enable cross-condition comparisons. Schematics were created with BioRender (https://biorender.com). **(B)** UMAP plot showing major immune cell populations integrated across CIDP, HC, and MS datasets, and **(C)** UMAP plot split by disease condition. **(D)** Dot plot for expression of canonical marker genes. **(E)** Heatmap of pairwise Pearson correlations between pseudo-bulk gene expression profiles of immune cell types. The distance metric (1 – Pearson’s r) was used for hierarchical clustering. Abbreviations used in the scRNA-seq data: B Memory, Memory B cell; B Naïve, Naïve B cell; CD4 Effector, CD4 Effector T cell; CD4 Memory, CD4 Memory T cell; CD4 Naïve, CD4 Naïve T cell; CD8 Effector, CD8 Effector T cell; CD8 Naïve, CD8 Naïve T cell.

To define the peripheral immune architecture of CIDP, we implemented a two-stage analytical framework integrating single-cell transcriptomic discovery with cohort-level validation (**Figure 1A**). Peripheral blood was obtained from 20 patients with CIDP and 20 age- and sex-matched healthy controls (HCs). For single-cell analysis, a discovery subset (CIDP, n = 2; HC, n = 2) with the highest RNA integrity was selected and integrated with publicly available PBMC scRNA-seq datasets from individuals with relapsing–remitting MS (n = 3; GSE133028) (14) and additional HCs (n = 3; GSE149689) (13), yielding a cross-disease immune reference framework. Given the limited sample size of the single-cell discovery subset, key transcriptomic observations were systematically validated in the full CIDP cohort using cytokine profiling and flow cytometry.

Participants with CIDP had a median age of 64 years, and eight patients (40%) were female, with no significant demographic differences between the CIDP and HC groups (**Table 1**). All samples were obtained during clinically stable periods under maintenance immunotherapy.

**Table 1:** Baseline characteristics of the study participants.

Variable	CIDP (n = 20)	HC (n = 20)	P value
Age, years, median (IQR)	64.2 (51.9 – 67.6)	58.0 (45.1 – 64.8)	0.211
Female, n (%)	8 (40.0)	13 (65.0)	0.113
Disease duration, years, median (IQR)	11.7 (5.1 – 17.6)	–	
Diabetes mellitus, n (%)	2 (10.0)	0 (0.0)	0.49
Treatment, n (%)			
Prednisolone	9 (45.0)	0 (0.0)	–
Immunosuppressant	17 (85.0)	0 (0.0)	–
mRS, median (IQR)	2.5 (1.0 – 4.0)	–	–
CIDP rating scale, median (IQR)			
INCAT	4.0 (1.0 – 5.3)	–	–
MRC sum score	56.0 (47.5 – 60.0)	–	–
I-RODS	35.0 (21.0 – 43.0)	–	–

CIDP, chronic inflammatory demyelinating polyneuropathy; HC, healthy control; INCAT, Inflammatory Neuropathy Cause and Treatment disability score; IQR, interquartile range; MRC, Medical Research Council; mRS, modified Rankin Scale; and I-RODS, Inflammatory Rasch-built Overall Disability Scale.

Following QC and normalization (Methods), high-resolution clustering with semi-supervised annotation referencing a PBMC atlas (18) identified 11 immune cell clusters (**Figure 1B**). Data quality was confirmed by standard QC metrics (**Supplementary Figures 1A–D**). UMAP visualization showed substantial overlap across CIDP, MS, and HC samples, indicating no global disease-driven segregation (**Figure 1C**). Canonical lineage markers validated cluster identities (**Figure 1D**), and pseudobulk correlations confirmed lineage-level transcriptomic consistency (**Figure 1E**). Minor variation in cell-type composition was observed (**Supplementary Figure 1E**); therefore, subsequent analyses focused on within-cell-type transcriptional differences rather than compositional shifts.

### CIDP shows enriched type I interferon and inflammasome activation

**Figure 2:**
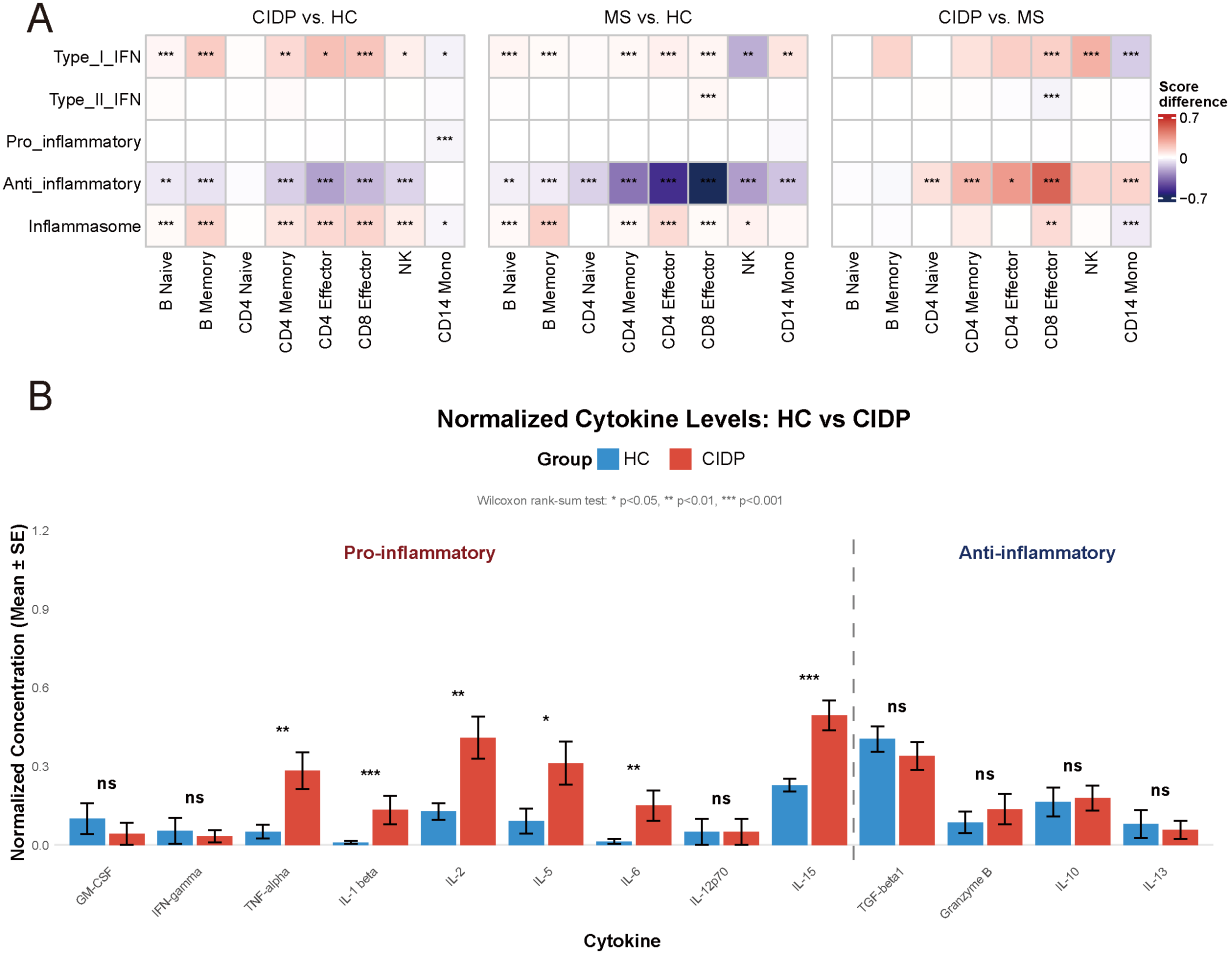
Distinct immune activation signatures between two demyelinating diseases. **(A)** Immune signature module scores. Module scores were computed per sample and cell type for five canonical immune programs—Type I interferon, Type II interferon, Pro-inflammatory, Anti-inflammatory, and Inflammasome—using predefined signature genes. Heatmaps display condition-wise differences in module scores (CIDP vs. HC, MS vs. HC, CIDP vs. MS); color encodes the direction and magnitude of the score difference. For each cell type and module, a Kruskal–Wallis test across the three groups (CIDP, HC, MS) assessed an overall effect, followed by Dunn’s *post hoc* tests for pairwise contrasts (e.g., CIDP vs. HC) with adjusted *P* values. **(B)** Cytokine profiles. Bar plot of normalized serum cytokine levels comparing healthy controls (HC, blue) and CIDP (red); values are min–max scaled (0–1). Bars represent mean ± SEM (HC *n* = 20, CIDP *n* = 20). Cytokines are grouped by function with a dashed divider: Pro-inflammatory cytokines: GM-CSF, IFN-γ, TNF-α, IL-1β, IL-2, IL-5, IL-6, IL-12p70, IL-15. Anti-inflammatory cytokines: TGF-β1, Granzyme B, IL-10, IL-13. For each cytokine, HC vs. CIDP was tested using Wilcoxon rank-sum test; significance: *P < 0.05, **P < 0.01, ***P < 0.001, ns not significant.

To delineate disease-associated inflammatory programs, we assessed innate and adaptive immune gene module scores across all cell types (**Figure 2A**; **Supplementary Table 2**; **Supplementary Figures 2A–E**). Both CIDP and MS exhibited broad inflammatory activation compared with HCs. However, CIDP showed preferential enrichment of type I interferon and inflammasome-related programs, whereas MS showed stronger suppression of anti-inflammatory pathways. Direct comparison between CIDP and MS revealed that this enrichment was most prominent in CD8 effector T cells (**Figure 2A**). Type II interferon signatures were largely unchanged overall, with an increase observed mainly in MS CD8 effector T cells.

These transcriptional patterns were evaluated in the full CIDP cohort using serum cytokine profiling. Patients with CIDP showed increased pro-inflammatory cytokines, including TNF-α, IL-1β, IL-2, IL-5, IL-6, and IL-15, while IFN-γ levels were comparable to those in HCs (**Figure 2B**). Notably, IL-1β, a canonical inflammasome-associated cytokine, was significantly elevated in CIDP, supporting heightened inflammasome activity. These findings define a CIDP-associated inflammatory profile characterized by type I interferon and inflammasome pathway activation.

### Shared and distinct pathway-level signatures in CIDP and MS

**Figure 3:**
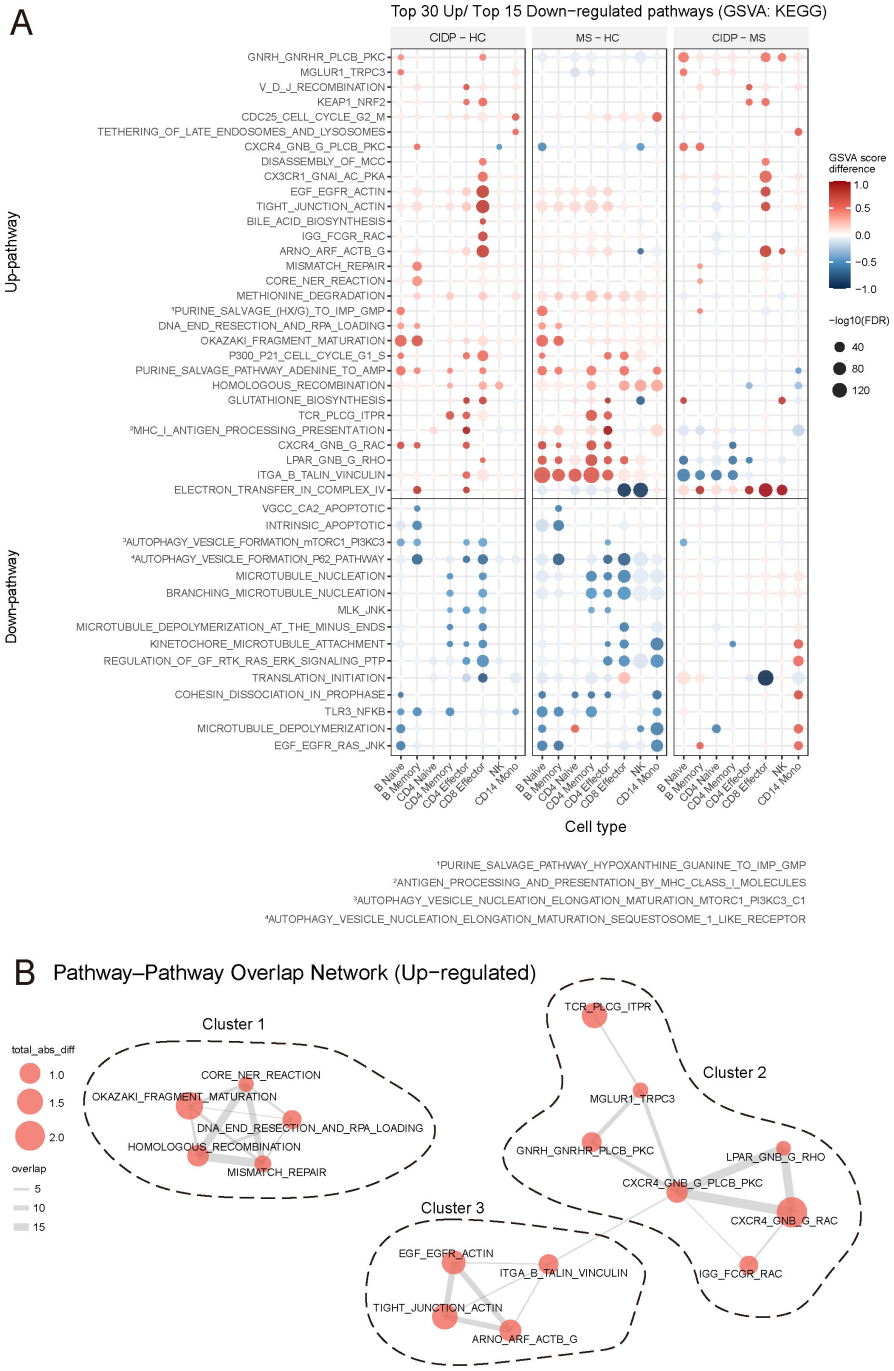
Cell-type–resolved distinct molecular pathways. **(A)** Top 30 up- and 15 down-regulated KEGG pathways derived from GSVA. Bubble plots summarize pathway activity per cell type across three contrasts (CIDP vs HC, MS vs HC, CIDP vs MS). Gene sets were taken from MSigDB (KEGG MEDICUS). For each contrast and cell type, GSVA scores were computed and the score difference was shown. Among the pathways ranked within CIDP−HC by the aggregate absolute median score difference across cell types, the top 30 up-regulated and top 15 down-regulated pathways were selected for visualization. Pathways (y-axis) are ordered by hierarchical clustering of their contrast-by-cell-type score profiles. Group differences were tested with Kruskal–Wallis across CIDP, HC, and MS, followed by Dunn’s *post-hoc* tests for pairwise comparisons. **(B)** Overlap networks constructed from the top 30 CIDP-upregulated pathways identified by GSVA. Nodes represent pathways, and edges link pathway pairs that share genes. Communities were outlined to highlight functionally coherent clusters. total_abs_diff, total absolute value difference.

Pathway-level analysis using GSVA identified distinct patterns of immune activation across CIDP and MS (**Figure 3A**). The top 30 upregulated and 15 downregulated pathways in CIDP relative to HCs were selected for functional clustering, which revealed three major groups (**Figure 3B**). Cluster 1 comprised DNA repair and replication–related pathways, reflecting proliferative or activation-associated transcriptional states (29, 30), with enrichment predominantly in B cells. Cluster 2 included broadly elevated receptor-mediated signaling pathways across multiple immune subsets. Cluster 3 encompassed actin-remodeling and cytoskeletal-organization pathways.

Although these pathway groups were generally upregulated in both CIDP and MS compared with HCs, the cytoskeletal module was most prominently enriched in CIDP CD8 effector T cells, indicating enhanced cytoskeletal organization and activation in this population. In contrast, MS showed marked enrichment of the integrin–talin–vinculin adhesion axis in B cells and CD4 T-cell subsets (**Figure 3A**). This pattern aligns with leukocyte adhesion and trafficking mechanisms targeted by natalizumab (31) and mirrors the limited benefit of VLA-4–directed strategies in peripheral demyelination (32).

These findings indicate that CIDP and MS share broad inflammatory activation but differ in pathway specialization, with CIDP showing prominent B-cell and CD8 effector T-cell involvement.

### CIDP B cells are numerically reduced but transcriptionally activated

**Figure 4:**
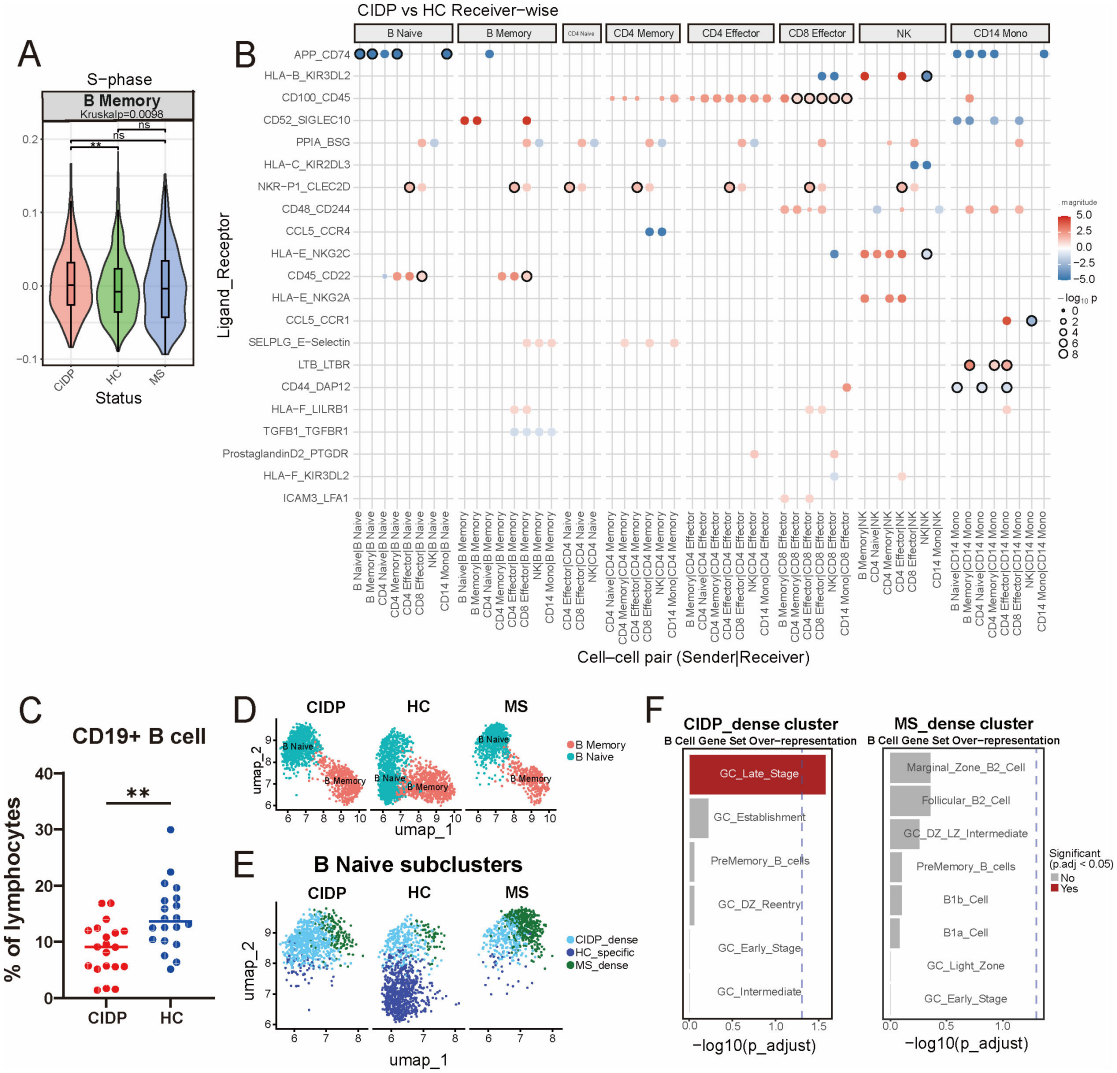
Distinct B cell activation dynamics in CIDP and MS. **(A)** Cell-cycle activity. S-phase scores were calculated with AddModuleScore using Seurat’s cc.genes.updated.2019. Within each cell type, Kruskal–Wallis tested group differences across CIDP, MS, HC followed by Dunn’s *post-hoc* pairwise tests with Bonferroni correction. **(B)** Cell–cell communication analysis comparing interaction features between CIDP and HC. Ligand–receptor interactions were inferred per donor with 1,000 permutations, and interactions with *P* < 0.05 were considered significant. For each sender–receiver pair, donors’ counts of significant versus non-significant interactions were compared between groups using two-sided Fisher’s exact tests on 2×2 contingency tables. Effect sizes were calculated as differences in proportions (n_si_g/*n_total_*) with Benjamini–Hochberg FDR correction. Heatmaps and bubble plots show proportion differences and aggregate mean interaction scores, highlighting alterations in intercellular communication between CIDP and HC. Bubble color indicates direction of change, and size reflects statistical significance (−log_10_*P*), using the smaller value when both groups are significant. Outlined bubbles denote significant interactions in both CIDP and HC. **(C)** CD19^+^ B-cell frequency within lymphocytes from flow cytometry analysis. Student’s *t*-test. **(D, E)** B-cell subclustering and enrichment. All B cells were isolated from integrated atlas and re-embedded, then reclustered to define discrete B Naïve and B Memory subclusters. Within the B Naïve compartment, several subclusters showed disease-biased distributions, including groups CIDP_dense, MS_dense and HC specific. **(F)** Over-representation analysis against a curated naïve-B gene set. Significance was based on the overlap between subcluster DEGs and the curated set (FDR < 0.05). Significance: *P < 0.05, **P < 0.01, ***P < 0.001, ns not significant.

We next examined whether pathway-level B-cell activation translated into altered cellular states and abundance (33). CIDP memory B cells exhibited elevated S-phase module scores (**Figure 4A**), indicating enhanced proliferative or activation-associated transcriptional activity. Consistent with this, cell–cell interaction analyses revealed increased CD45–CD22 engagement among B cells, particularly within the naïve B-cell compartment, in CIDP (**Figure 4B**; **Supplementary Figures 3A, B**); CD45–CD22 is a regulatory axis that modulates B-cell receptor activation thresholds and signaling sensitivity (34).

Flow cytometric analysis showed a marked reduction in total B-cell frequencies in CIDP compared with HCs (**Figure 4C**), whereas T-cell proportions were preserved or increased. FoxP3+ regulatory T-cell proportions did not differ significantly between CIDP and HC groups (**Supplementary Figure 4**). In contrast, the regulatory B-cell proportions were also significantly decreased (**Supplementary Figure 4**), suggesting impaired counter-regulatory capacity and a shift toward a more activation-prone B-cell landscape.

To further delineate disease-specific B-cell states, we subclustered naïve B cells and identified CIDP-, MS-, and HC-enriched subpopulations (**Figures 4D, E**). The CIDP-enriched cluster displayed upregulation of germinal center (GC)–associated genes (**Figure 4F**), together with metabolic pathways supporting nucleotide biosynthesis and cellular activation (**Supplementary Figure 5A**). In contrast, MS B cells exhibited an activation signature without consolidation into a distinct GC-associated state (**Supplementary Figure 5B**). When interpreted in the context of metabolic features and state-defining gene sets (26–28), these findings suggest that CIDP B cells are numerically reduced but transcriptionally activated, with features overlapping those of GC-associated activation states (27, 35).

### CIDP CD8 effector T cells exhibit cytotoxicity-related and cytoskeletal transcriptional signatures

**Figure 5:**
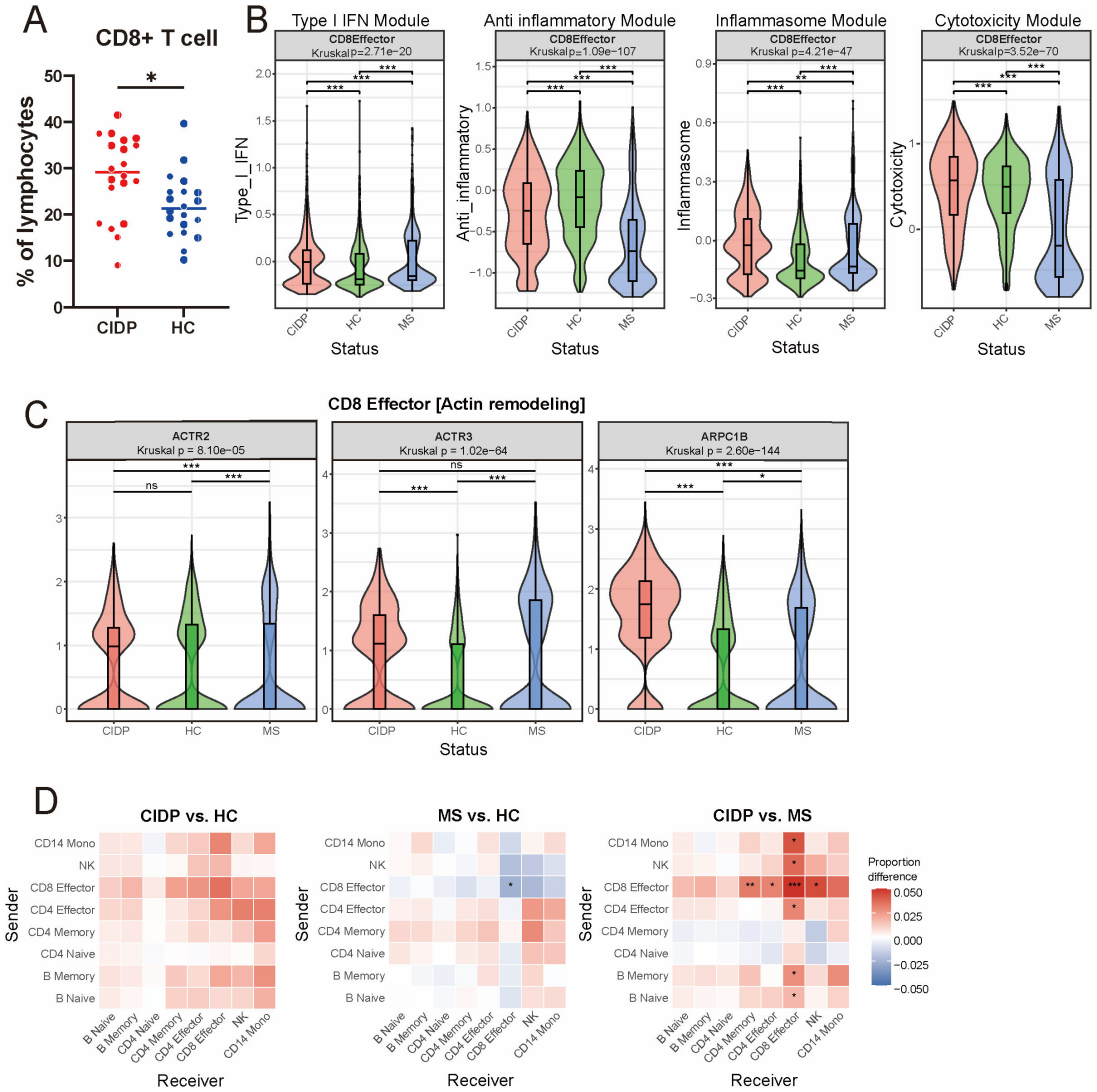
Distinct T cell activation dynamics between CIDP and MS. **(A)** CD8^+^ T cell frequency within lymphocytes from flow cytometry analysis. Student’s *t*-test **(B)** Immune signature module scores in CD8^+^ effector. For each signature within CD8^+^ effectors, Kruskal–Wallis test followed by Dunn’s *post-hoc* tests with Bonferroni adjustment were applied. (Type I IFN, Anti-inflammatory, Inflammasome, Cytotoxicity module) **(C)** Core-gene expression from Cluster 3 in **Figure 3B**. Genes recurrently shared across pathways in this cluster (e.g., ACTR2, ACTR3, ARPC1B) were designated core genes, and their expression was visualized as violin plots across CIDP, MS, and HC. Kruskal–Wallis tests followed by Dunn’s *post-hoc* tests with Bonferroni correction were applied. **(D)** Global Cell–Cell Ligand–Receptor interaction intensity. For each sender-receiver pair, the difference in the proportion of significant interactions between groups were presented. Group differences were tested with two-sided Fisher’s exact tests on counts of significant vs. non-significant interactions, with Benjamini–Hochberg FDR correction. Color encodes direction and magnitude (red = higher in the left group; blue = higher in the right group); Asterisks indicate significant interactions based on FDR values. In CIDP vs. MS, CD8^+^ effector T cells show a clear increase in interaction intensity (as both sender and receiver), indicating strengthened CD8-centric communication in CIDP. Significance: *P < 0.05, **P < 0.01, ***P < 0.001, ns not significant.

Given the enrichment of actin-remodeling pathways in CIDP CD8 effector T cells, we next assessed whether these transcriptional features reflected heightened cytotoxic activation (**Figures 3A, B**). Flow cytometry analyses revealed an increased proportion of CD8 T cells among total lymphocytes in CIDP compared with HCs (**Figure 5A**). Within CD8 effector clusters, CIDP exhibited elevated module scores for type I interferon signaling, inflammasome activation, and cytotoxicity, alongside suppression of anti-inflammatory programs (**Figure 5B**).

To dissect the cytoskeletal component of this activation, we examined genes shared across the enriched actin-remodeling pathways. Several members of the ARP2/3 complex emerged as recurrently represented, prompting further evaluation of *ACTR2 (ARP2), ACTR3 (ARP3)*, and *ARPC1B* expression (**Figure 5C**). *ACTR2* showed no significant difference between CIDP and HCs but was elevated relative to MS, whereas *ACTR3* was increased in CIDP compared with both HCs and MS. *ARPC1B* exhibited striking and selective upregulation in CIDP relative to both groups. As ARP2/3 complex components are essential for IL-2–driven T-cell activation, immunological synapse formation, and cytotoxic effector function (36), selective *ARPC1B* upregulation underscores a distinctive cytoskeletal remodeling feature of CD8 effector T cells in CIDP.

Cell–cell interaction analysis further demonstrated disease-specific remodeling of the intercellular communication network (**Figure 5D**). CIDP CD8 effector T cells exhibited increased interaction intensity relative to MS across multiple ligand–receptor pairs. In particular, the CD100–CD45 axis, a promoter of T-cell proliferation, motility, and activation (37), was more prominently engaged in CIDP (**Figure 4B**), whereas the opposite trend was observed in MS (**Supplementary Figures 3A, B**). These interaction patterns parallel the elevated circulating IL-15 levels observed in CIDP (**Figure 2B**), a cytokine involved in CD8 T-cell maintenance, proliferation, and cytotoxic activation (38).

Together, these findings suggest that CIDP is associated with increased CD8 T-cell representation at the cohort level and cytotoxicity-related transcriptional features within CD8 effector T cells in the scRNA-seq discovery analysis. Because direct functional assays were not performed, these data should be interpreted as evidence of an effector transcriptional program rather than proof of enhanced cytotoxic activity.

## Discussion

By integrating single-cell transcriptomics with cohort-level immunophenotyping, we identify a coordinated peripheral immune architecture in CIDP that is distinct from MS. Our findings converge on two principal immune axes: dysregulated B-cell states characterized by reduced cell numbers despite persistent activation, and a prominent cytotoxic CD8 T-cell program supported by cytoskeletal remodeling and intercellular signaling. Together, these axes provide an integrative framework for understanding immune dysregulation in CIDP.

Our findings refine the immunological contrast between CIDP and MS. MS is classically characterized by exaggerated type II interferon (IFN-γ–driven) responses (39), and our analyses similarly showed modest increases of type II IFN signatures in MS CD8 effector T cells. In contrast, CIDP demonstrated a pronounced skew toward type I IFN activation, together with upregulated inflammasome-related cytokines such as IL-1β. This cytokine pattern was validated in the full cohort, which showed consistent elevation of pro-inflammatory cytokines. Clinical observations further reinforce this pattern: IFN-α exposure has been associated with CIDP onset (40), and IFN-β demonstrated no therapeutic benefit in a randomized CIDP trial (41), highlighting that interferon-targeted strategies do not translate directly between MS and CIDP. Collectively, these findings support a type I IFN–inflammasome–skewed inflammatory program in CIDP, distinct from the type II IFN–dominant responses observed in MS.

Within this broader inflammatory context, B-cell dysregulation represents a central component of the CIDP immune architecture. In MS, IL-10-producing transitional and regulatory B-cell subsets exhibit impaired IL-10-mediated control of Th1 responses (42), while T follicular helper cells promote B-cell maturation and intrathecal antibody production within the CNS (43). The clinical efficacy of B-cell-depleting therapies in MS underscores this central role. Furthermore, MS B cells and CD4 T cells in our analyses showed activation of integrin-dependent adhesion and trafficking programs involving the integrin–talin–vinculin axis, which is aligned with the mechanism targeted by natalizumab (44). In contrast, CIDP showed limited enrichment of these trafficking-related pathways, with no clear evidence for a VLA-4–dependent migratory program comparable to that observed in MS. Instead, CIDP exhibited a pattern defined by reduced B-cell abundance coupled with transcriptional activation and depletion of regulatory B cells. Given the role of regulatory B cells in restraining effector T-cell responses (45, 46), their reduction may contribute to the enhanced cytotoxic and inflammatory features observed in CIDP CD8 effector T cells. These findings align with the partial clinical efficacy of B-cell–directed therapies reported in refractory CIDP, including rituximab, ocrelizumab (47), and ofatumumab (48), suggesting that B-cell dysregulation in CIDP is mechanistically distinct from the trafficking-driven pathology observed in MS.

Beyond numerical loss, CIDP B cells demonstrated transcriptional enrichment of germinal center-associated programs, including metabolic pathways supporting ATP production, purine metabolism, and nucleotide biosynthesis (26–28). These transcriptional and metabolic features are consistent with activation states overlapping GC-associated programs (27, 35), indicating that despite numerical contraction, the residual B-cell compartment remains functionally active. This apparent paradox of depletion with persistent activation may reflect several non-mutually exclusive mechanisms, including treatment-related modulation of the circulating B-cell compartment, redistribution of activated B cells to affected tissues, and selective enrichment of activated GC-like B-cell subsets within the residual circulating B-cell population. Together, these mechanisms may contribute to impaired immune regulation and sustained downstream effector responses.

Notably, the cytotoxic CD8 T-cell axis emerged as a particularly prominent feature in CIDP, accompanied by enrichment of actin-remodeling pathways and increased expression of ARPC1B, a key regulator of immunological synapse formation and cytotoxic T-cell function (36). Because ARPC1B dysregulation has been implicated in immune disorders (49), its increased expression may reflect an activation-associated cytotoxic state. Whether this finding is specific to CIDP or represents a more general feature of T-cell activation will require direct functional validation.

Cell–cell interaction analyses further support this model by revealing an expanded intercellular signaling network sustaining CD8 effector activation in CIDP. The engagement of the CD100–CD45 axis (37) and elevated circulating IL-15 levels (38) together suggest that cytotoxic CD8 effector activity is maintained through both intrinsic activation and extrinsic signaling cues. This coordinated activation contrasts with the comparatively lower interaction intensity observed in MS CD8 effector T cells, further emphasizing disease-specific immune organization.

From a therapeutic perspective, these findings highlight key distinctions between peripheral and central demyelinating diseases. The limited enrichment of integrin-mediated adhesion pathways in CIDP, compared with MS, is consistent with the modest efficacy of trafficking-targeted therapies (32), and indicates that alternative immune mechanisms likely play a more prominent role in CIDP pathogenesis. Instead, the convergence of type I interferon signaling, dysregulated B-cell states, and cytotoxic CD8 T-cell activation points toward a broader immune network underlying the heterogeneous and often incomplete responses to current immunotherapies in CIDP. Targeting pathways downstream of type I interferon signaling or modulating cytotoxic T-cell effector function may therefore represent candidate therapeutic strategies, although these require further validation in mechanistic and clinical studies.

This study has several limitations. First, the single-cell analysis was performed in a small discovery subset, reflecting the rarity of CIDP and the challenges of obtaining high-quality samples; these findings should therefore be interpreted as exploratory. Second, the analysis was restricted to peripheral blood rather than nerve or CSF compartments. Third, the MS reference data were derived from publicly available datasets with different sample preparation protocols, and cross-dataset comparisons may be influenced by technical variability despite batch correction. Fourth, although patients were clinically stable at sampling, all CIDP samples were obtained under maintenance immunotherapy. Thus, the observed immune signatures cannot be attributed solely to untreated CIDP biology and may reflect residual disease-related immune activity and treatment effects. Fifth, we did not perform direct functional validation of the inferred B-cell activation-related state, CD8 cytotoxicity-related transcriptional program, or type I IFN/inflammasome-related signatures. Future studies should incorporate larger, longitudinal, and treatment-stratified cohorts, with functional immune assays.

In summary, we identified CIDP-associated peripheral immune signatures that differed from those observed in the MS reference dataset, including dysregulated B-cell states and CD8 effector T-cell activation. This dual immune axis, embedded within a type I IFN– and inflammasome-skewed inflammatory context, may provide an exploratory framework for understanding the heterogeneous therapeutic responses observed in CIDP and suggest the B cell–CD8 effector axis as a potential target for disease-specific immunomodulation. These findings warrant further validation in future larger, longitudinal, and treatment-stratified studies.

## Data Availability

The datasets presented in this study can be found in online repositories. The names of the repository/repositories and accession number(s) can be found below: http://kbds.re.kr/, KSX10000018, KSX10000019, KSX10000020, and KSX10000021.

## Supplementary material

Supplementary Figure 1 Single-cell RNA-seq quality metrics and cell-type composition. **(A)** Violin plots showing the number of genes per cell (nFeature_RNA), number of UMI per cell (nCount_RNA), percent of reads mapped to mitochondrial reads per cell (% Mitochondrial Genes), and percent of reads aligned to ribosomal reads per cell (% Ribosomal RNA) by batch. **(B)** Cell-type composition across disease conditions (CIDP, HC, MS).B Memory, Memory B cell; B Naïve, Naïve B cell; CD4 Effector, CD4 Effector T cell; CD4 Memory, CD4 Memory T cell; CD4 Naïve, CD4 Naïve T cell; CD8 Effector, CD8 Effector T cell; CD8 Naïve, CD8 Naïve T cell.

Supplementary Figure 2 Immune landscape module scoring. Immune signature module scores across all cell types stratified by disease condition. **(A)** Type I interferon module score, **(B)** Type II interferon module score, **(C)** Pro-inflammatory module score, **(D)** Anti-inflammatory module score, and **(E)** Inflammasome module score.

Supplementary Figure 3 Ligand–receptor interaction between disease groups (MS-HC, CIDP-MS). **(A)** Cell–cell communication analysis comparing interaction features between CIDP and MS. Ligand–receptor interactions were inferred per donor with 1,000 permutations, and interactions with *P* < 0.05 were considered significant. For each sender–receiver pair, donors’ counts of significant versus non-significant interactions were compared between groups using two-sided Fisher’s exact tests on 2×2 contingency tables. Effect sizes were calculated as differences in proportions (*n*_si_g/*n*_total_) with Benjamini–Hochberg FDR correction. Heatmaps and bubble plots display proportion differences and aggregate mean interaction scores, highlighting alterations in intercellular communication between CIDP and MS. Bubble color indicates the direction of change, and size reflects statistical significance (−log_10_*P*), using the smaller value when both groups are significant. Outlined bubbles denote interactions significant in both CIDP and MS. **(B)** Cell–cell communication analysis as in (A), comparing MS and HC.

Supplementary Figure 4 Flow cytometry analysis of regulatory T and B cell proportions. **(A)** Gating strategy for identifying regulatory T cell lineages. **(B)** Proportion of regulatory T cells among CD4 T cells in CIDP and HC. **(C)** Proportion of CD4 T cells among lymphocytes. **(D)** Gating strategy for analyzing regulatory B cell lineages. **(E)** Proportion of regulatory B cells among B cells in CIDP and HC. Student’s *t*-test. Significance: **P* < 0.05, ns not significant.

Supplementary Figure 5 Altered B cell repertoire in disease conditions. **(A)** Gene Ontology Biological Process (GO: BP) enrichment analysis of DEGs from naïve B cell subclusters (CIDP_dense, MS_dense, and HC_specific). The top 15 significantly enriched GO terms for CIDP_dense are shown, with a dashed line marking the significance threshold (*P* = 0.05). **(B, C)** GO: BP enrichment analysis as in **(A)**, for the MS_dense **(B)** and HC_specific **(C)** subclusters. **(D)** Venn diagram showing the overlap of DEGs among naïve B cell subclusters (CIDP_dense, MS_dense, and HC_specific). **(E)** Heatmap of the top 10 DEGs specific to each naïve B cell subcluster (CIDP_dense, MS_dense, and HC_specific).

Supplementary Table 1 Patient information.

Supplementary Table 2 Immune module scoring gene list.

Supplementary Table 3 Cytokine raw levels.

Supplementary Table 4 Flow cytometry antibody.
