# Supplementary material for: Integrated peripheral immune profiling reveals B-cell dysregulation and CD8 effector signatures in chronic inflammatory demyelinating polyneuropathy
Source: Front Immunol. 2026 Jul 24;17:1888882. doi: 10.3389/fimmu.2026.1888882 (PMC13448232; doi:10.3389/fimmu.2026.1888882)
Supplement: Supplementary file 1 [file Table3.docx]

**Supplementary Table 3. Cytokine raw levels**

|  | CIDP | HC | P value | FDR |
| --- | --- | --- | --- | --- |
| Pro-inflammatory cytokines (pg/mL) | | | |  |
| GM-CSF | 0.77 ± 3.43 | 1.83 ± 4.79 | 0.602 | 0.857 |
| IFN-γ | 0.86 ± 2.65 | 1.40 ± 5.83 | 0.989 | >0.999 |
| TNF-α | 163.41 ± 180.85 | 29.09 ± 67.79 | **0.003** | **0.013** |
| IL1-β | 11.31 ± 20.58 | 0.76 ± 2.21 | **0.001** | **0.006** |
| IL2 | 11.84 ± 9.88 | 4.15 ± 3.88 | **0.007** | **0.021** |
| IL5 | 1.01 ± 1.18 | 0.29 ± 0.69 | **0.043** | 0.093 |
| IL6 | 527.10 ± 904.79 | 46.53 ± 139.92 | **0.008** | **0.021** |
| IL12-p70 | 0.05 ± 0.24 | 0.05 ± 0.24 | >0.999 | >0.999 |
| IL15 | 2.09 ± 1.08 | 0.97 ± 0.46 | **<0.001** | **0.006** |
| Anti-inflammatory cytokines (pg/mL) | | | |  |
| TGF-β | 149476 ± 38358 | 159770 ± 34658 | 0.253 | 0.470 |
| Granzyme B | 3.26 ± 6.21 | 2.06 ± 4.45 | 0.659 | 0.857 |
| IL10 | 9.49 ± 11.29 | 8.70 ± 13.14 | 0.602 | 0.857 |
| IL13 | 3.18 ± 8.57 | 4.37 ± 13.16 | 0.989 | >0.999 |

CIDP: chronic inflammatory demyelinating polyneuropathy, HC: healthy control
