# Supplementary material for: Integrated peripheral immune profiling reveals B-cell dysregulation and CD8 effector signatures in chronic inflammatory demyelinating polyneuropathy
Source: Front Immunol. 2026 Jul 24;17:1888882. doi: 10.3389/fimmu.2026.1888882 (PMC13448232; doi:10.3389/fimmu.2026.1888882)
Supplement: Supplementary file 2 [file Table4.docx]

**Supplementary Table 4. Antibody panels used for flow cytometry**

| **Assay** | **Commercial reagent** | **Marker** | **Clone** | **Fluorochrome** | **Manufacturer** |
| --- | --- | --- | --- | --- | --- |
| **Regulatory B-cell analysis** | DuraClone IM B Cells Tube (Cat. No. B53318) | IgD | IA6-2 | FITC | Beckman Coulter |
|  |  | CD21 | BL13 | PE | Beckman Coulter |
|  |  | CD19 | J3-119 | ECD | Beckman Coulter |
|  |  | CD27 | 1A4CD27 | PC7 | Beckman Coulter |
|  |  | CD24 | ALB9 | APC | Beckman Coulter |
|  |  | CD38 | LS198-4-3 | APC-A750 | Beckman Coulter |
|  |  | IgM | SA-DA4 | Pacific Blue | Beckman Coulter |
|  |  | CD45 | J33 | Krome Orange | Beckman Coulter |
| **Regulatory T-cell analysis** | DuraClone IM Treg Tube (Cat. No. B53346) | CD45RA | 2H4LDH11LDB9 (2H4) | FITC | Beckman Coulter |
|  |  | CD25 | B1.49.9 | PE | Beckman Coulter |
|  |  | CD39 | BA54 | PC5.5 | Beckman Coulter |
|  |  | CD4 | SFCI12T4D11 (T4) | PC7 | Beckman Coulter |
|  |  | FoxP3 | 259D | Alexa Fluor 647 | Beckman Coulter |
|  |  | CD3 | UCHT-1 | APC-A750 | Beckman Coulter |
|  |  | Helios | 22F6 | Pacific Blue | Beckman Coulter |
|  |  | CD45 | J33 | Krome Orange | Beckman Coulter |

APC, allophycocyanin; APC-A750, allophycocyanin Alexa Fluor 750; ECD, phycoerythrin-Texas Red-X; FITC, fluorescein isothiocyanate; PC5.5, phycoerythrin-Cyanine 5.5; PC7, phycoerythrin-Cyanine 7.
